# Predictors of Vasculitis in Patients With Systemic Lupus Erythematosus in Bangladesh

**DOI:** 10.7759/cureus.80868

**Published:** 2025-03-20

**Authors:** Shadab Saud Sunny, Mohammad Abul Kalam Azad, M Masudul Hassan, ATM Asaduzzaman, Md Nazrul Islam

**Affiliations:** 1 Rheumatology, Comilla General Hospital, Comilla, BGD; 2 Rheumatology, Bangabandhu Sheikh Mujib Medical University (BSMMU), Dhaka, BGD; 3 Dermatology, Bangabandhu Sheikh Mujib Medical University (BSMMU), Dhaka, BGD

**Keywords:** cutaneous vasculitis, flare of lupus, lupus flare, lupus vasculitis, rheumatoid arthritis, sle, sle and lupus nephritis, vasculitis, vasculitis and disease activity

## Abstract

Introduction

Systemic lupus erythematosus (SLE) is a multisystem autoimmune disease. Sometimes vasculitis may complicate the disease. Among the manifestations of SLE, vasculitis presentation is common. This study aimed to identify the predictors of vasculitis in SLE patients.

Methods

This is a cross-sectional study in a single center conducted in the Department of Rheumatology, Bangabandhu Sheikh Mujib Medical University (BSMMU), Dhaka from December 2019 to January 2021. The disease activity and damage were assessed using the Systemic Lupus Erythematosus Disease Activity Index (SLEDAI) and Systemic Lupus International Collaborating Clinics/American College of Rheumatology damage index (SLICC/ACR DI). Study subjects were grouped into vasculitis and no vasculitis groups. The multivariate logistic regression analysis was done to determine the independent predictors of vasculitis in SLE. The *p*-value <0.05 was considered significant.

Results

The rate of lupus vasculitis was 14.3%. The significant difference in vasculitis features between vasculitis and no vasculitis groups were: acute cutaneous lupus erythematosus (ACLE) (p<0.001), oral ulcer (p<0.001), alopecia (p<0.001), Raynaud’s phenomenon (p=0.011), fever (p=0.002), arthritis (p<0.001), pregnancy loss (p=0.003), lupus nephritis (p=0.032), seizure (p=0.027), pleurisy (p=0.027), leucopenia (p=0.049), anti-dsDNA positivity (p=0.008), hypocomplementemia (p=0.003), higher mean SLEDAI (p<0.001) and SLICC/ACR DI score (p<0.001). In multivariate logistic regression analysis, higher SLEDAI score (OR = 1.296, 95% CI =1.114-1.508) was positively and lupus nephritis (OR= 0.055, 95% CI =0.007-0.413) was negatively associated with lupus vasculitis.

Conclusion

The vasculitis flare of lupus is associated with Raynaud’s phenomenon and pregnancy loss. Mucocutaneous flare, fever, arthritis, seizure, pleurisy, and lupus nephritis were also associated with vasculitis.

## Introduction

This article abstract was presented as a poster at APLAR conferences held on 28-31 August 2021 and at the EULAR congress held on 1-4th June Copenhagen and in IRACON 21-24th Nov. Bangalore. Autoimmunity with multisystem [1] involvement is the cardinal feature of systemic lupus erythematosus (SLE) and it is usually diverse [2]. The prevalence [3] of vasculitis in SLE may vary up to 50%. Lupus vasculitis (LV) may present with mild skin involvement to severe organ involvement. The characteristic pattern of cutaneous vasculitis [4] is leukocyte infiltration in the vessel wall results in endothelial injury and fibrinoid necrosis. Involvement of the hands and feet is frequent in cutaneous small vessel vasculitis (CSVV) and is the most common form of vasculitis in patients with SLE with a prevalence of 10% [5]. The CSVV [5] may present as a punctate erythematous lesion, purpura, periungual infarction, ischemic ulcerated lesion, urticaria, tender red macule or papule, splinter hemorrhage, and nodule. Lupus may present as medium vessel vasculitis (MVV) [6] manifested as mononeuritis multiplex, mesenteric vasculitis, and digital gangrene. Vasculitis involving large vessels is rare in lupus patients, with limited case reports [7]. In the previous studies, the reported associated factors of LV were very diverse. It is reported that LV is associated with acute cutaneous rash, oral ulcer, alopecia, leucopenia, and fever. Among them, the most common one is the malar rash. Discoid rash, Raynaud’s phenomenon, lupus nephritis, antiphospholipid syndrome (APS), myositis, anemia, leucopenia, hypocomplementemia, high disease activity, and higher and Systemic Lupus International Collaborating Clinics/American College of Rheumatology (SLICC/ACR) damage index are also reported association of LV [8]. There are published reports [9] that younger age at onset, the longer disease duration, male sex, myocarditis, psychosis, serositis, lymphopenia, pleuritis, high fever, raised ESR, anti-La antibodies, Coombs positivity, anti-Smith ab, and anti-RNP positivity, seizure, musculoskeletal manifestations, anti-ribosomal P protein antibody, anti-Ro antibodies are present similar to that of the other musculoskeletal diseases as LV, reports [10] showed that it has prognostic importance as the occurrence of it is usually associated with disease flare, organ damage and fatality. Identification of associated factors and proper treatment may delay or halt future damage, and save lives. This study aimed to identify the predictors of vasculitis in SLE patients attending a tertiary care hospital.

## Materials and methods

This cross-sectional study was conducted in the Lupus Clinic of Bangladesh located in the Department of Rheumatology, Bangabandhu Sheikh Mujib Medical University (BSMMU), Dhaka, Bangladesh. Patients from all corners of Bangladesh are referred to this clinic. Ethical clearance was obtained from the Institutional Review Board (IRB) of BSMMU (Approval number: BSMMU/2019/13905). The study period was from December 2019 to January 2021. Data were collected systematically by semi-structured questionnaire. The calculated sample size was more than 154 (n>50+8m, at 5% level and 80% power) and we enrolled 168 consecutive SLE patients, who fulfilled the 2019 ACR/EULAR classification criteria. Patients with rheumatoid arthritis, systemic sclerosis, dermatomyositis, mixed connective tissue disease (MCTD) or overlap, malignancy, diabetes mellitus, infective endocarditis, and chronic hepatitis were excluded from the study. The study was performed following the Declaration of Helsinki principles and after having the informed written consent. All patients were evaluated with detailed history and clinical examination for the presence of malar rash, generalized maculopapular rash, subacute cutaneous lupus erythematosus (SCLE), chronic cutaneous lupus erythematosus (CCLE), oral ulcer, alopecia, Raynaud’s phenomenon, arthritis, fever, lupus nephritis, seizure, psychosis, transverse myelitis, pleurisy, pericarditis, Avascular necrosis (AVN), pulmonary hypertension and the manifestations of APS. Antinuclear antibodies (ANA) analysis was performed using immunofluorescence on Hep-2 cells during the recruitment of patients. Following laboratory tests were done during the study visit: complete blood count with ESR, routine urinalysis, serum creatinine, alanine aminotransferase (ALT), anti-dsDNA, serum C3, C4, anti-phospholipid antibodies. Anti-dsDNA was measured using the ELISA method, titer value of more than 75 U/ml was considered positive [11]. Complement levels were estimated by the nephelometric system, a C3 value less than 0.9 g/l and/or C4 value less than 0.1 g/l were considered as low complement. All patients were evaluated for the occurrence of vasculitis features like palpable purpura, erythematous punctate lesions, tender erythematous macule or papule, tender finger nodule, ulcer, cutaneous nodule, digital gangrene, mesenteric vasculitis, and mononeuritis multiplex. The presence of cutaneous vasculitis was confirmed clinically by a dermatologist by biopsy findings. Vasculitic digital gangrene was diagnosed by clinical examination and duplex ultrasound with biopsy findings consistent with vasculitis.

Mesenteric vasculitis was confirmed by abdominal CT scan and probable cases were diagnosed by the presence of typical clinical features and exclusion of peptic ulcer disease, acute pancreatitis, acute gastroenteritis, peritonitis, and other causes of acute abdomen by history, clinical examination, abdominal ultrasound, and endoscopy of the upper gastrointestinal tract. Vasculitic neuropathy was diagnosed clinically and by nerve conduction study and was confirmed by biopsy. The disease activity and damages were assessed using SLEDAI and SLICC/ACR DI.

Statistical analysis

All the data were checked after collection. Data were processed and analyzed using SPSS (Statistical Package for Social Sciences), version 25. The prevalence of vasculitis was expressed in number and percentage. Socio-demographic, clinical, laboratory, disease activity, and damage indices were compared between the two groups. The chi-square, Fisher exact, Student’s t-test, and Mann-Whitney U test were used where found appropriate. The multivariate logistic regression analysis was done to determine the independently associated predictors of vasculitis in SLE. AP-value<0.05 was considered significant. The odd ratio was set at a 95% CI.

## Results

A total of 208 patients were screened for enrollment in the Lupus Clinic of BSMMU. Among them, 40 patients were excluded for not fulfilling the 2019 ACR/EULAR classification criteria of SLE, inclusion criteria, and not coming back with requested reports. After exclusion, a total of 168 SLE patients were enrolled. LV was observed in 24 (14.3%) patients. Among the patients with vasculitis, 16 (66.7%), 8 (33.3%), and 1 (4.16%) had SVV, MVV, and both, respectively. Palpable purpura was a common manifestation of 11 (68.8%) SVV, followed by 9 (56.3%) tender erythematous macule or papule, 5 (31.3%) erythematous punctate lesions, 1 (6.3%) leg ulcer, 1 (6.3%) cutaneous nodule, and 1 (6.3%) bullous lesion. Among eight patients with MVV, 3 (37.5%) had mesenteric vasculitis, 3 (37.5%) had vasculitis neuropathy, and 2 (25%) had digital gangrene. Patients with lupus MVV had a long duration of disease (7±4.5 vs. 3.2±3.3 years, p = 0.014) and high SLICC/ACR damage index (1.50±1.195vs.0.69±0.704,p=0.023) compared to lupus SVV. Patients with lupus SVV had frequent ACLE: acute cutaneous lupus erythematosus (ACLE) 15 (93.8%) vs. 4 (50.0%) than MVV and that was statistically significant (p = 0.014). The mean age of the LV and without vasculitis group was 25.7±6.6 and 26.7±7.6 years, respectively. In the LV group, all patients were female, 134 (93.1%) without vasculitis group were female. Overweight and obesity were observed in 6 (25%) and 73 (50.7%) patients with LV and without vasculitis, respectively. The mean age at onset of symptoms was 21.3±6 years vs. 22.1±6.9 years in LV vs. without vasculitis. The mean duration of disease was 4.5±4.1 and 4.6±4.2 years respectively in the LV and without vasculitis group. ACLE, oral ulcer, alopecia, Raynaud’s phenomenon, fever, arthritis, lupus nephritis, seizure, pleurisy, and pregnancy loss were significantly associated with LV. Comparisons of clinical features of SLE patients with vasculitis and without vasculitis are shown in Table 1.

**Table 1:** Comparison of clinical features of lupus vasculitis and without vasculitis (n=168) *Fisherʼs eхact test ^γ^chi-square test, p ≤ 0.05 is considered statistically significant ^β^all diagnosed cases of lupus nephritis, presented with or without flare SD: standard deviation; n: number; %: percent; ACLE: acute cutaneous lupus erythematosus; SCLE: subacute cutaneous lupus erythematosus; CCLE: chronic cutaneous lupus erythematosus; DVT: deep vein thrombosis; APS: anti-phospholipid antibody syndrome; AVN: avascular necrosis; HTN: hypertension

Clinical Features	Lupus Vasculitis (n=24)	Without Vasculitis (n=144)	P-value
	n (%)	n (%)	
Fever	13 (54.2)	36 (25.0)	0.002^γ^
SLE-specific skin lesions			
ACLE	19 (79.2)	27 (18.8)	0.000^γ^
SCLE	1 (4.2)	6 (4.2)	0.500*
CCLE	0 (0)	9 (6.3)	
SLE non-specific skin lesions			
Oral ulcer	17 (70.8)	19 (13.2)	0.000^γ^
Alopecia	20 (83.3)	39 (27.1)	0.000^γ^
Palpable purpura	12 (50.0)	0 (0)	
Thrombocytopenic purpura	0 (0)	2 (1.4)	
Leg ulcer	1 (4.2)	2 (1.4)	0.186*
Cutaneous nodule	1 (4.2)	0 (0)	
Erythematous punctate lesion	6 (25.0)	0 (0)	
Bulla	1 (4.2)	0 (0)	
Raynaud’s phenomenon	5 (20.8)	8 (5.6)	0.011*
Arthritis	17 (70.8)	43 29.9)	0.000^γ^
Lupus nephritis^β^	6 (25.0)	65 (45.1)	0.032^γ^
Neuro psychiatric			
Seizure	2 (8.3)	1 (0.7)	0.027*
Psychosis	1 (4.2)	2 (1.4)	0.186*
Transverse myelitis	0	2 (1.4)	
Serositis			
Pleurisy	2 (8.3)	1 (0.7)	0.027*
Pericarditis	1 (4.2)	3 (2.1)	0.231*
Fetal loss	11 (45.8)	35 (24.3)	0.014^γ^
1 fetal loss	5 (20.8)	22 (15.3)	
2 fetal loss	5 (20.8)	6 (4.2)	
3 or more fetal loss	1 (4.2)	7 (4.9)	
No loss	13 (54.2)	109 (75.7)	
DVT	1 (4.2)	6 (4.2)	0.500*
APS	3 (12.5)	11 (7.6)	0.213*
AVN	1 (4.2)	2 (1.4)	0.186*
Pulmonary HTN	2 (8.3)	3 (2.1)	0.074*

The laboratory features of SLE patients with vasculitis and without vasculitis are shown in Table 2. Patients with vasculitis were associated with a higher SLICC/ACR damage index. Pulmonary hypertension, AVN, loss, and resection of digits and toes, myocarditis, pancreatitis, venous stasis, peripheral neuropathy and muscle atrophy, extensive alopecia, and scarring were observed in LV patients. The SLEDAI score was positively associated with LV (OR=1.296, 95% CI=1.114-1.508) and lupus nephritis was negatively associated with LV (OR=0.055, 95% CI=0.007-0.413) (Table 3). ACLE, oral ulcer, Raynaud’s, and alopecia although had high OR, they were not statistically significant.

**Table 2:** Comparison of laboratory features of Lupus vasculitis and without vasculitis (n=168) ^γ^chi-Square test *Fisherʼs eхact test ^λ^Student’s t-test, p < 0.05 is considered statistically significant SD: standard deviation; n: number; %: percent; anti-dsDNA: antibody against double-strand DNA; ACLE: acute cutaneous lupus erythematosus; SLEDAI: SLE Disease Activity Index; SLICC-ACR DI: Systemic Lupus International Collaborating Clinics - American College of Rheumatology Damage Index a: Anemia defined according to WHO, b: Leucopenia defined as total leucocyte count less than 3000/mm^3^, c: Lymphopenia defined as absolute lymphocyte count less than 1000/mm^3^, d: Thrombocytopenia defined as a total platelet count less than 1,00,000/mm^3^, e: Low C3/ Low C4/ Both

Laboratory Features	Lupus Vasculitis (n=24) n(%)	Without Vasculitis (n=144) n(%)	Odds Ratio	95% CI Lower	95% CI Upper	P-value
Anaemia^a^	20 (83.3)	113 (78.5)	0.729	0.232	2.290	0.293^γ^
Leucopenia^b^	2 (8.3)	2 (1.4)	0.155	0.021	1.157	0.049*
Lymphopenia^c^	7 (29.2)	29 (20.1)	0.612	0.232	1.615	0.159^γ^
Thrombocytopenia^d^	0	7 (4.9%)				
ESR (mean±SD)	59.8±43.7	48.1±31.7				0.058^λ^
Anti-dsDNA positivity	21 (87.5)	90 (62.5)	0.238	0.068	0.836	0.008^γ^
Low Complement^e^	21 (87.5)	85 (59)	0.206	0.059	0.722	0.003^γ^
Antiphospholipid antibody positivity (Ever)	9/13 (69.2)	15/35 (42.9)				0.052^γ^
SLEDAI (mean±SD)	21.3±7.6	7.6±6.9				<0.001^λ^
SLICC/ACR DI (mean±SD)	0.9±0.9	0.3±0.6				<0.001^λ^

**Table 3:** Multiple logistic regression analysis of protocol-based factors significant in univariate analysis Anti ds-DNA: Antibody against double-strand DNA; SLEDAI: SLE Disease Activity Index; SLICC-ACR DI: Systemic Lupus International Collaborating Clinics - American College of Rheumatology Damage Index

Variable	Odds Ratio	95% CI		P-value
		Lower	Upper	
Fever	0.222	0.035	1.433	0.114
ACLE	2.562	0.309	21.223	0.383
Oral ulcer	3.418	0.560	20.876	0.183
Alopecia	1.449	0.258	8.130	0.673
Raynaud’s phenomenon	2.142	0.243	18.876	0.493
Arthritis	0.833	0.112	6.203	0.858
Lupus nephritis	0.055	0.007	0.413	0.005
Anti-dsDNA positivity	0.526	0.071	3.878	0.528
Low complement a	0.991	0.128	7.698	0.993
SLEDAI	1.296	1.114	1.508	0.001
SLICC/ACR DI	1.898	0.646	5.577	0.244

## Discussion

The prevalence of LV in this study was 14%. The reported prevalence of LV varies globally. In a cohort from Cairo [12], the prevalence was 33.45, in Brazil [13] the reported prevalence of cutaneous vasculitis was 15.4%, and in Egypt [14] 14.8%. A Lower prevalence of 12.5% of LV was reported in India [9].

In the LV group, all patients were female, 134 (93.1%), it was similar in previous studies [15] where 89% were female.

LV especially cutaneous vasculitis was associated with a cutaneous flare of SLE. The Birmingham [16] SLE cohort showed that high disease activity is associated with cutaneous vasculitis similar to this study and LV had an increased association with malar rash, we didn’t find any association between DLE and LV, which may be for infrequent visits of DLE patients in our OPD. We found an association between seizures with LV and a similar association had been reported in vasculitis is associated with seizure by Ramsey-Goldman et al. [17]. We also found an association of pleurisy with LV. Different pulmonary associations were observed by Drenkard et al. [18].

Hypocomplementemia was more frequent in patients with LV than without vasculitis. Complement deposition and complement consumption play a major role in its pathogenesis in LV has been reported in previous studies [19]. Similar findings are seen in this study where an association between vasculitis with low C3 was observed. Faridin et al.’s study [20] found that anti-dsDNA positivity was significantly associated with LV, but, some of our LV patients were negative for anti-dsDNA even at the flare. It indicates that there may be different pathogenesis in coetaneous LV. LV was associated with not only cutaneous flare, but also with arthritis, fever, seizure, anti-ds- DNA positivity, and hypocomplementemia. So, LV was a major manifestation of flare lupus. We found that patients with LV had more pregnancy losses. Previous studies showed flares of SLE result in poor pregnancy outcomes [21] and we have found a novel finding that LV is associated with poor pregnancy outcomes. In our study, LV was associated with arthritis and leucopenia as such it may be assumed that LV may be a manifestation of APS. The frequency of antiphospholipid antibody positivity was higher in the LV group compared to SLE without the vasculitis group (69.2% vs. 42.9%) in this study. Cutaneous manifestations of APS [22] resemble the manifestations of vasculitis and both conditions can co-exist.

In this study, CSVV presented as palpable purpura was the common manifestation. Mesenteric vasculitis, vasculitic neuropathy, and digital gangrene were uncommon. Patients with MVV had a long duration of disease and a higher SLICC/ACR damage index compared to cutaneous LV. Patients with cutaneous LV had frequent ACLE. A similar observation had been described in previous studies [18,23].

There are several limitations. This was a hospital-based cross-sectional study, which may not reflect the true predictors of LV in the community. Diagnosis of LV was mostly based on clinical features. A retrospective search of vasculitis was done using clinical files. No therapy data regarding vasculitis was available. Due to resource constraints, we failed to do the biopsy and some essential laboratory tests in all cases. Due to logistic constraints, antineutrophil cytoplasmic autoantibody (ANCA) status could not be measured. Current and past medication history was not considered as time constraints due to the COVID-19 pandemic. We have thoroughly examined the patients and reviewed the records. We have checked many determinants in a single study. This was the strength of our study.

## Conclusions

Vasculitis in SLE is life-threatening. Arthritis, seizure, pleurisy, fever, lupus nephritis, and mucocutaneous manifestations are associated with LV. Besides these manifestations, Raynaud’s phenomenon, pregnancy loss, and high mean SLICC/ACRDI score are also associated with the development of vasculitis in patients with SLE. Early prediction of flare may save lives.

## References

[1] Lahita RG, Tsokos G, Buyon J, Koike T (2010). Systemic lupus erythematosus. 5th Edition.

[2] Rahman A, Isenberg DA (2008). Systemic lupus erythematosus. N Engl J Med.

[3] Leone P, Prete M, Malerba E, Bray A, Susca N, Ingravallo G, Racanelli V (2021). Lupus vasculitis: An overview. Biomedicines.

[4] Cassisa A, Cima L (2024). Cutaneous vasculitis: Insights into pathogenesis and histopathological features. Pathologica.

[5] Lotti T, Ghersetich I (1998). Cutaneous small-vessel vasculitis. J Am Acad Dermatol.

[6] Kumar N, Choudhary N, Agarwal G, Rizvi Y, Kaul B, Ahlawat R (2007). Extensive medium-vessel vasculitis with SLE: An unusual association. J Clin Rheumatol.

[7] Böckle BC, Jara D, Aichhorn K, Junker D, Berger T, Ratzinger G, Sepp NT (2014). Cerebral large vessel vasculitis in systemic lupus erythematosus. Lupus.

[8] Kallas R, Goldman D, Petri MA (2020). Cutaneous vasculitis in SLE. Lupus Sci Med.

[9] Mathur R, Deo K, Raheja A (2022). Systemic lupus erythematosus in India: A clinico-serological correlation. Cureus.

[10] Toubi E, Kessel A, Bamberger E, Golan TD (2004). Systemic lupus erythematosus vasculitis: A current therapeutic overview. Curr Treat Options Cardiovasc Med.

[11] Rouquette AM, Desgruelles C (2006). Detection of antibodies to dsDNA: An overview of laboratory assays. Lupus.

[12] Gamal SM, Mohamed SS, Tantawy M, Siam I, Soliman A, Niazy MH (2021). Lupus-related vasculitis in a cohort of systemic lupus erythematosus patients. Arch Rheumatol.

[13] das Chagas Medeiros MM, Bezerra MC, Braga FN (2015). Clinical and immunological aspects and outcome of a Brazilian cohort of 414 patients with systemic lupus erythematosus (SLE): Comparison between childhood-onset, adult-onset, and late-onset SLE. Lupus.

[14] Shahin AA, Zayed HS, Elrefai RM (2018). The distribution and outcome of vasculitic syndromes among Egyptians: A multi-center study including 630 patients. Egyptian Rheumatol.

[15] Bertsias GK, Pamfil C, Fanouriakis A, Boumpas DT (2013). Diagnostic criteria for systemic lupus erythematosus: Has the time come?. Nat Rev Rheumatol.

[16] Yee CS, Su L, Toescu V (2015). Birmingham SLE cohort: Outcomes of a large inception cohort followed for up to 21 years. Rheumatology (Oxford).

[17] Ramsey-Goldman R, Alarcón GS, McGwin G (2008). Time to seizure occurrence and damage in PROFILE, a multi-ethnic systemic lupus erythematosus cohort. Lupus.

[18] Drenkard C, Bao G, Dennis G, Kan HJ, Jhingran PM, Molta CT, Lim SS (2014). Burden of systemic lupus erythematosus on employment and work productivity: Data from a large cohort in the southeastern United States. Arthritis Care Res.

[19] Gheita TA, Abaza NM, Sayed S, El-Azkalany GS, Fishawy HS, Eissa AH (2018). Cutaneous vasculitis in systemic lupus erythematosus patients: Potential key players and implications. Lupus.

[20] Faridin HP, Megawaty AA, Adnan E (2022). The correlation between the levels of anti-dsDNA IgA antibody and the severity of systemic lupus erythematosus based on cutaneous vasculitis. Med Clín Práct.

[21] Phansenee S, Sekararithi R, Jatavan P, Tongsong T (2017). Pregnancy outcomes among women with systemic lupus erythematosus: A retrospective cohort study from Thailand. Lupus.

[22] Lally L, Sammaritano LR (2015). Vasculitis in antiphospholipid syndrome. Rheum Dis Clin North Am.

[23] Ramos-Casals M, Nardi N, Lagrutta M (2006). Vasculitis in systemic lupus erythematosus: Prevalence and clinical characteristics in 670 patients. Medicine.

